# RadioComics—Santa Claus and the breakthrough reaction

**DOI:** 10.1186/s13244-024-01835-0

**Published:** 2024-10-16

**Authors:** Paolo Lombardo, Knud Nairz, Ingrid Boehm

**Affiliations:** Department of Diagnostic, Interventional, and Pediatric Radiology, Inselspital, University of Bern, Bern, Switzerland

## Abstract

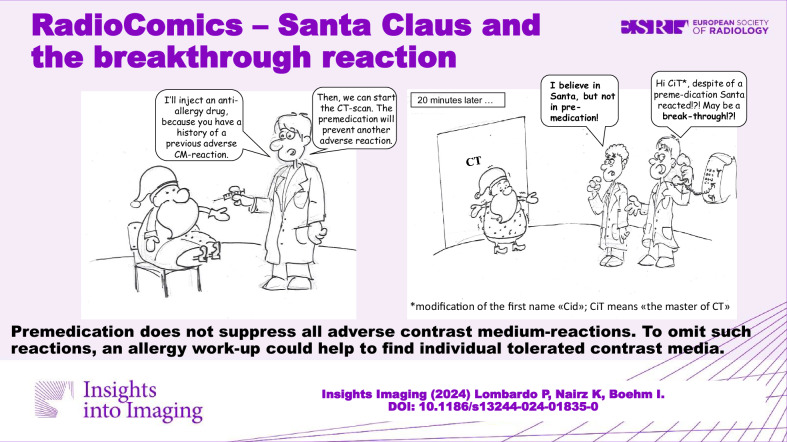

Image-guided examinations are required by all medical disciplines and for a majority of patients [1]. Many imaging challenges need to be enhanced, and every year, several 100 million doses of contrast media (iodinated > gadolinium-based) are applied worldwide. Fortunately, most patients tolerate the administration of a contrast medium (CM) well, and only a minority, approximately 2%, of patients exhibit adverse reactions in the form of hypersensitivity [2]. Patients with a history of mild or moderate reactions (such as urticaria, itching, angioedema, slight shortness of breath, and tachycardia, for example) are at increased risk of reacting again. Traditionally, in such patients, prophylaxis is performed as anti-allergic medication with a corticosteroid only or as a combination of corticosteroid plus H1 antihistamine. This premedication before the next CM-enhanced imaging is intended to reduce the frequency of CM-hypersensitivity reactions in patients at risk [3]. However, premedication targets adverse symptoms only, not the cause of allergy, and therefore is not always effective. If premedication fails, allergic symptoms are still observable and are termed a “breakthrough reaction” [4, 5]. We could show that they occur significantly more often when the documented diagnoses are inexact or sloppy [6]. We recently tried a fundamentally different approach to communicating the topic of CM-associated adverse effects and invented a new category of comics [7, 8], which we term radiocomics. Here, we present a radiocomics addressing breakthrough reactions because, in the context of CM, they are largely unknown (Fig. 1).

**Fig. 1:**
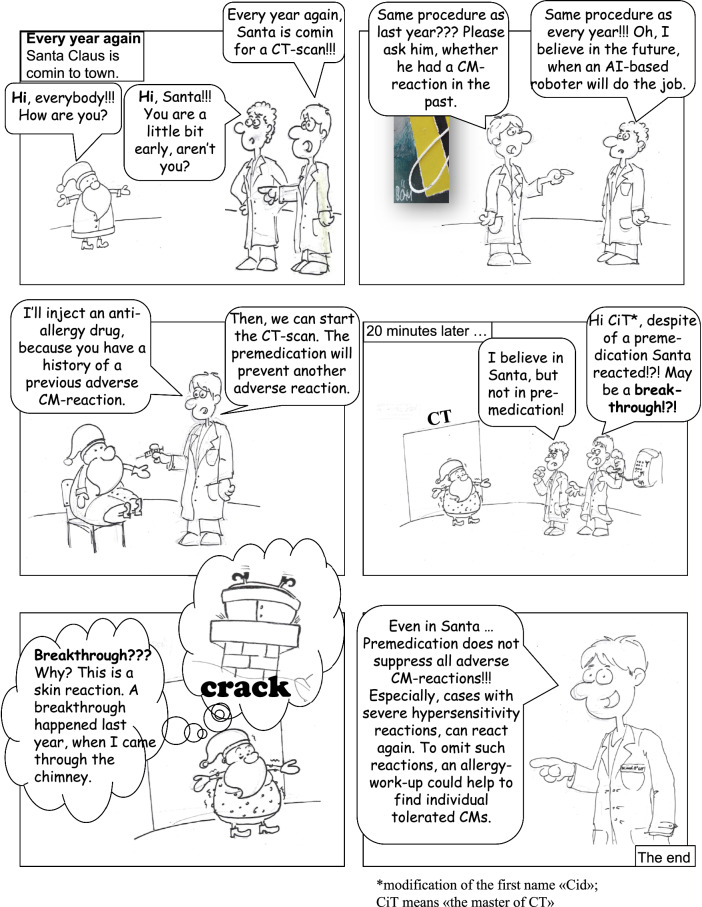
RadioComics - Santa Claus and the Breakthrough Reaction

## Data Availability

Not applicable.
